# Prevalence of Comorbid Hyperparathyroidism and Its Association with Renal Dysfunction in Asian Patients with X-Linked Hypophosphatemic Rickets/Osteomalacia

**DOI:** 10.1007/s00223-025-01359-9

**Published:** 2025-03-12

**Authors:** Nobuaki Ito, Hee Gyung Kang, Toshimi Michigami, Noriyuki Namba, Takuo Kubota, Ayumi Shintani, Ryota Kawai, Daijiro Kabata, Haruka Ishii, Yayoi Nishida, Seiji Fukumoto, Keiichi Ozono

**Affiliations:** 1 Division of Therapeutic Development for Intractable Bone Diseases, Graduate School of Medicine and Faculty of Medicine, The University of Tokyo, 7-3-1 Hongo, Bunkyo-Ku, Tokyo, 113-8655 Japan; 2 Osteoporosis Center, The University of Tokyo Hospital, Tokyo, Japan; 3 Division of Pediatric Nephrology, Department of Pediatrics, Seoul National University Children’s Hospital, Seoul, South Korea; 4 Department of Bone and Mineral Research, Osaka Women’s and Children’s Hospital, Osaka Prefectural Hospital Organization, Osaka, Japan; 5 Division of Pediatrics and Perinatology, Faculty of Medicine, Tottori University, Tottori, Japan; 6 Department of Pediatrics, Osaka University Graduate School of Medicine, Osaka, Japan; 7 Department of Medical Statistics, Osaka Metropolitan University School of Medicine, Osaka, Japan; 8 Center for Mathematical and Data Sciences, Kobe University, Hyogo, Japan; 9 Medical Affairs Department, Kyowa Kirin Co., Ltd, Tokyo, Japan; 10 Tamaki-Aozora Hospital, Tokushima, Japan; 11 Center for Promoting Treatment of Intractable Diseases, ISEIKAI International General Hospital, Osaka, Japan

**Keywords:** Chronic kidney disease, Fibroblast growth factor 23, Hyperparathyroidism, Hypophosphatemia, Parathyroid hormone, X-linked hypophosphatemic rickets/osteomalacia

## Abstract

In patients with X-linked hypophosphatemic rickets/osteomalacia (XLH) in Asia, the current prevalence of hyperparathyroidism and its association with renal dysfunction have not been determined. We used patient data retrospectively collected up to the time of informed consent in the SUNFLOWER study, a long-term observational study, to investigate the current treatment status and prevalence of comorbid hyperparathyroidism and its association with renal dysfunction in patients with XLH in Japan and South Korea. Of 69 patients who met the eligibility criteria, 32 (46.4%) did not have hyperparathyroidism (hereinafter referred to as non-hyperparathyroidism), 33 (47.8%) had secondary hyperparathyroidism, and four (5.8%) had tertiary hyperparathyroidism. Men were more prone to develop secondary and tertiary hyperparathyroidism, use oral phosphate at higher frequencies, and have a higher incidence of Stage ≥ 3 chronic kidney disease and Grade ≥ 3 renal calcification than women. Ongoing treatments for patients with XLH and non-hyperparathyroidism, secondary hyperparathyroidism, and tertiary hyperparathyroidism mainly consisted of active vitamin D (30 [93.8%], 25 [75.8%], and 3 [75.0%], respectively) and oral phosphate (21 [65.6%], 23 [69.7%], and 4 [100.0%], respectively). At informed consent, patients with tertiary hyperparathyroidism had the lowest estimated glomerular filtration rate values. Our study highlights the prevalence of comorbid hyperparathyroidism and its association with renal dysfunction in patients with XLH through a large-scale observational study in Asia.

Trial registration: NCT03745521; UMIN000031605.

**Supplementary Information:**

The online version contains supplementary material available at 10.1007/s00223-025-01359-9.

## Introduction

X-linked hypophosphatemic rickets/osteomalacia (XLH) is an X-linked dominantly inherited rare disease presenting with persisting hypophosphatemia and impaired bone mineralization due to excessive fibroblast growth factor 23 (FGF23) caused by loss-of-function variants in the phosphate-regulating gene with homologies to endopeptidases on the X chromosome *(PHEX)* gene [1–5]. The estimated incidence of XLH in Japan was reported to be 1 in 20,000 live births from 2005 to 2009 [6]. FGF23 is a regulator specific to serum phosphate concentration. It lowers the serum phosphate concentration via two mechanisms: one is the inhibition of phosphate reabsorption in the renal proximal tubules, and the second is reduced intestinal absorption of phosphate due to reduced 1,25-hydroxyvitamin D [1,25(OH)_2_D] concentration caused by the inhibition of renal 1α-hydroxylase activity and acceleration of 24-hydroxylase activity [7].

Many patients with XLH develop rickets because of chronic hypophosphatemia and impaired mineralization of growth plate chondrocytes in their childhood. This results in skeletal abnormalities (for example, curved lower limbs), growth disorders, short stature continuing into adulthood, gait abnormality, and dental symptoms [1, 2]. Adult patients with XLH have increased bone pain and risk of bone fracture and develop arthropathy, enthesopathy, and spinal ligament ossification [1, 2]. Patients with XLH presenting limb deformity may need surgical correction [8–11]. Regarding management strategies of children and adults with XLH, a practice guide in the United States [1], guidelines in Europe [5], a consensus in the Asia Pacific [3], and diagnostic criteria in Japan [12] have been published. However, there are no internationally unified guidelines.

Until recently, XLH was treated with active vitamin D and oral phosphate several times daily. However, these conventional treatments cannot normalize the serum phosphate concentrations of the patients and do not address the further elevated FGF23 concentrations due to positive feedback from conventional therapy [1, 13]. Furthermore, long-term use of these treatments might cause secondary hyperparathyroidism, tertiary hyperparathyroidism, nephrocalcinosis, and the development and progression of chronic kidney disease (CKD). The risk of secondary and tertiary hyperparathyroidism in patients with XLH has been reported in several small- to large-scale clinical studies in the past [1, 14–22]. However, in patients with XLH in Japan and other parts of Asia, the current prevalence of hyperparathyroidism and its association with renal dysfunction have not yet been well determined. We initiated a long-term observational study called the SUNFLOWER (Study of longitUdinal observatioN for patients with X-Linked hypOphosphatemic rickets/osteomalacia in collaboration With Asian partnERs) study, in Japan and South Korea in 2018, to collect information on the disease course and treatment of patients with XLH over time [23]. The present study aimed to investigate the current treatment status and prevalence of comorbid hyperparathyroidism and its association with renal dysfunction in patients with XLH in Japan and South Korea. The analysis used patient information at the time of informed consent in the SUNFLOWER study and before informed consent was obtained.

## Materials and Methods

### Study Design

The SUNFLOWER study is a prospective, longitudinal, observational cohort study of patients with XLH. Enrollment began on April 1, 2018, and closed on April 30, 2022. The present study is an observational study that obtained and analyzed data retrospectively collected up to the time of participation from patients enrolled in the SUNFLOWER study between April 1, 2018, and December 31, 2019. The detailed methodology of the SUNFLOWER study has been published elsewhere [23].

In the SUNFLOWER study, 226 patients diagnosed with XLH were enrolled: 172 in Japan and 54 in South Korea. The study was initiated on April 1, 2018, and the observational period will be from the time of enrollment to April 1, 2028, for a maximum of 10 years. To obtain overall data about XLH, there were no selection or exclusion criteria based on specific disease stages or severity.

The SUNFLOWER study is being conducted in Japan and South Korea in compliance with the latest version of the Declaration of Helsinki and all relevant national regulations in Japan and South Korea. Ethics committees from the principal investigator’s medical institution and Kyowa Kirin Co., Ltd. approved the study protocol and consent forms. In participating medical institutions, their ethics committee also gave their approval. The study was registered in “clinical study database (UMIN)” established by the National University Hospital Council of Japan and in “ClinicalTrials.gov.” under the respective identifiers: NCT03745521; UMIN000031605.

### Patients

The present study used data at the time of enrollment of 69 patients 18 years of age or older (55 patients in Japan and 14 in South Korea) selected from patients enrolled in 20 institutions (17 institutions in Japan and 3 in South Korea) between April 1, 2018, and December 31, 2019, in the SUNFLOWER study. The main inclusion criteria were as follows: Patients who met any of (1) to (3) criteria: (1) had a confirmed *PHEX* gene mutation; (2) had a consanguineous family member with an X-linked genetic relationship who had a confirmed *PHEX* gene mutation; or (3) whose intact FGF23 (Minaris Medical, Tokyo, Japan) was found to be more than 30 pg/mL with concomitant low serum phosphate level. Together with the above criteria, patients who presented with physical findings of rickets such as bone deformities (i.e., genu varum/valgum), abnormal spinal curvature, craniotabes, open fontanels, rachitic rosary, or joint swelling; clinical symptoms of osteomalacia (i.e., muscle weakness or bone pain); as well as laboratory findings pointing to either condition (e.g., decreased serum phosphate, low 1,25(OH)_2_D and increased bone-specific alkaline phosphatase levels) were included [12]. Patients who were 18 years of age or older and provided consent in writing by themselves were included. A cut-off level was selected for intact FGF23 (> 30 pg/mL) based on the study by Endo et al. [24].

Patients who were participating in another clinical study (trial) when consent was obtained and patients who were considered unsuitable for participation in the present study were excluded. For patient safety, those participating in a clinical study (trial) other than the present study could participate in the present study after their participation in the clinical study (trial) was completed.

### Study Endpoints and Variables

Patient background was evaluated based on age, sex, body height, weight, renal ultrasound, and laboratory assessments (blood and spot urine) when informed consent was obtained. In principle, random blood and urine sampling was performed at least 4 h after a meal or intake of a phosphate preparation (including for TmP/GFR assessment). Blood collection in the morning was preferred. Treatment status was based on data about the use of drugs for the primary disease and its complications, as well as surgery for the primary disease and its complications. Regarding the prevalence of comorbid hyperparathyroidism, hyperparathyroidism was classified according to intact parathyroid hormone (iPTH) and corrected calcium when informed consent was obtained, and history of parathyroidectomy for tertiary hyperparathyroidism in electronic data capture as follows: non-hyperparathyroidism (iPTH < 65 pg/mL); secondary hyperparathyroidism (iPTH ≥ 65 pg/mL and corrected calcium < 10.2 mg/dL); and tertiary hyperparathyroidism (iPTH ≥ 65 pg/mL and corrected calcium ≥ 10.2 mg/dL or a history of parathyroidectomy). These classifications were based on criteria from previous studies [22, 25], and the reference values of iPTH and corrected calcium were provided by SRL Inc., the company that performed the biochemical tests [26, 27]. Corrected calcium was calculated using the following formula: corrected calcium (mg/dL) = calcium (mg/dL) – serum albumin (g/dL) + 4.0 [28].

Nephrocalcinosis was assessed by renal ultrasound and using the following 5-grade system: Grade 0: normal, Grade 1: mild echogenicity around renal pyramid borders, Grade 2: severe echogenicity around renal pyramid borders and minor echogenicity of entire renal pyramids, Grade 3: uniformly severe echogenicity of entire renal pyramids, and Grade 4: calculus (echogenicity indicating a solitary lesion at the tip of renal pyramids) [29, 30].

The formula for calculating the estimated glomerular filtration rate (eGFR) is as follows [31]: eGFR (mL/min/1.73 m^2^) = 194 × serum creatinine(Cr)^−1.094^ × Age^−0.287^ (× 0.739 if a woman). The formula used to calculate tubular reabsorption of phosphate (TRP) is as follows: 1 – (serum Cr concentration (mg/dL) × urine P concentration (mg/dL))/(serum P concentration (mg/dL) × urine Cr concentration (mg/dL)). The formulas for tubular maximum phosphate reabsorption capacity (TmP/GFR) are as follows: TmP/GFR (mg/dL) = TRP × serum phosphate (mg/dL) [if TRP ≤ 0.86] and TmP/GFR (mg/dL) = 0.3 × TRP/(1 – (0.8 × TRP)) × serum phosphate (mg/dL) [if TRP > 0.86]. CKD definitions were as follows: Stage 1, eGFR ≥ 90 mL/min/1.73 m^2^; Stage 2, eGFR 60–89 mL/min/1.73 m^2^; Stage 3, eGFR 30–59 mL/min/1.73 m^2^; Stage 4, eGFR 15–29 mL/min/1.73 m^2^; and Stage 5, eGFR < 15 mL/min/1.73 m^2^ [32].

### Statistical Analysis

Descriptive statistics were calculated for background data after the patients were classed by hyperparathyroidism status. For continuous variables, the median and interquartile range were used, and for nominal variables, the proportion and frequency were used. To evaluate the association between hyperparathyroidism status and renal dysfunction, multivariable linear regression analysis was performed with eGFR as an outcome variable and the status of hyperparathyroidism as an explanatory variable. The analysis was adjusted for sex, age, and nationality. A two-sided significance level of 0.05 was adopted for hypothesis testing, and all analyses were performed using R (v4.03).

## Results

### Patient Characteristics

**Fig. 1:**
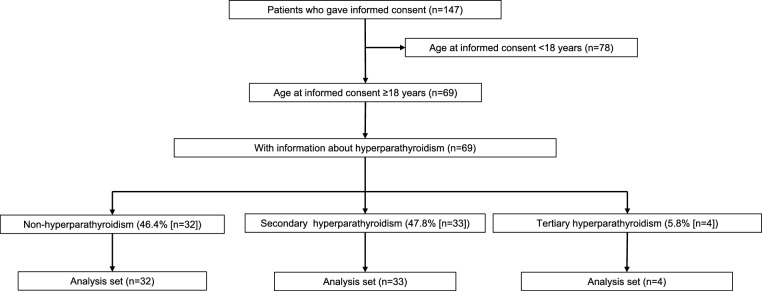
Disposition of patients with XLH and hyperparathyroidism. *XLH* X-linked hypophosphatemic rickets/osteomalacia

Figure 1 shows the patient disposition. Of 69 patients with XLH who met the inclusion criteria and provided informed consent, information about hyperparathyroidism was available for all 69 patients. Of the 69 patients, 32 (46.4%) patients did not have hyperparathyroidism; 33 (47.8%) had secondary hyperparathyroidism; and 4 (5.8%) had tertiary hyperparathyroidism. Table 1 summarizes patient background data by classification of hyperparathyroidism. Overall, patients had a median (interquartile range [IQR]) age of 34.0 [23.0, 48.0] years, 20.3% were from South Korea, and 34.8% were men. Median Z scores of height and weight were − 2.20 [− 2.72, − 1.18] and − 0.50 [− 1.28, 0.45], respectively, with a median Z score of body mass index of 0.66 [− 0.14, 1.36]. *PHEX* mutation was confirmed in 17 (53.1%) patients with non-hyperparathyroidism, 17 (51.5%) patients with secondary hyperparathyroidism, and 2 (50.0%) patients with tertiary hyperparathyroidism. In patients with non-hyperparathyroidism, secondary hyperparathyroidism, and tertiary hyperparathyroidism, respectively, median iPTH [IQR] was 45.00 [28.50, 52.00], 87.00 [75.20, 119.00], and 104.00 [50.25, 178.25] pg/mL; median corrected Ca [IQR] was 8.80 [8.60, 9.10], 8.80 [8.60, 9.10], and 8.80 [8.70, 8.97] mg/dL; TmP/GFR was 1.79 [1.56, 2.14], 1.43 [1.19, 1.69], and 1.65 [1.47, 1.69] mg/dL; and FGF23 level was 134.00 [88.00, 295.75], 168.00 [114.00, 427.00], and 562.50 [414.75, 1441.00] pg/mL.

**Table 1:** Characteristics of patients with XLH

	Overall	Non-hyperparathyroidism	Secondary hyperparathyroidism	Tertiary hyperparathyroidism
n (%)	69 (100)	32 (46.4)	33 (47.8)	4 (5.8)
Age, years, median [IQR]	34.00 [23.00, 48.00]	30.00 [21.75, 45.50]	34.00 [29.00, 48.00]	45.00 [35.75, 53.75]
Nationality, South Korea, n (%)	14 (20.3)	8 (25.0)	6 (18.2)	0 (0)
Sex, men, n (%)	24 (34.8)	9 (28.1)	13 (39.4)	2 (50)
Height, cm, median [IQR]	150.40 [144.98, 156.45]	151.80 [145.90, 156.50]	148.00 [143.90, 155.60]	159.05 [150.85, 162.12]
Height, Z-score, median [IQR]	− 2.20 [− 2.72, − 1.18]	− 2.05 [− 2.51, − 0.94]	− 2.41 [− 3.63, − 1.56]	− 2.00 [− 2.98, − 0.91]
Weight, kg, median [IQR]	52.10 [47.03, 59.05]	51.40 [46.50, 57.20]	53.20 [47.70, 59.20]	52.10 [48.27, 59.60]
Weight, Z-score, median, [IQR]	− 0.50 [− 1.28, 0.45]	− 0.52 [− 1.33, 0.36]	− 0.47 [− 1.09, 0.69]	− 0.64 [− 1.73, 0.40]
Body mass index, kg/m^2^, median [IQR]	23.00 [20.58, 25.63]	22.90 [20.45, 25.25]	22.90 [20.45, 25.25]	20.15 [19.85, 23.67]
Body mass index, Z-score, median [IQR]	0.66 [− 0.14, 1.36]	0.61 [− 0.20, 1.28]	0.73 [0.09, 1.83]	− 0.32 [− 0.44, 0.45]
*PHEX* mutation, n (%)	36 (52.2)	17 (53.1)	17 (51.5)	2 (50)
Age at diagnosis, years, median [IQR]	14.46 [1.38, 34.91]	11.46 [1.41, 37.01]	19.35 [1.45, 35.13]	0.37 [0.19, 1.43]
Time since diagnosis, years, median [IQR]	14.08 [1.70, 28.37]	13.18 [3.06, 20.60]	10.84 [1.37, 29.17]	50.63 [38.36, 55.07]
Ongoing treatment, n (%)				
Oral phosphate	48 (69.6)	21 (65.6)	23 (69.7)	4 (100.0)
Active vitamin D	58 (84.1)	30 (93.8)	25 (75.8)	3 (75.0)
History of calcimimetics				
Cinacalcet, n (%)	2 (2.9)	0 (0.0)	1 (3.0)	1 (25.0)
Evocalcet, n (%)	0 (0.0)	0 (0.0)	0 (0.0)	0 (0.0)
Parathyroidectomy	4 (5.8)	0	0	4 (100)
Serum phosphate, mg/dL, median [IQR] Reference value: 2.4–4.3 mg/dL	2.10 [2.00, 2.50]	2.25 [2.00, 2.55]	2.00 [1.80, 2.40]	2.40 [2.22, 2.58]
Serum calcium, mg/dL, median [IQR]	9.40 [9.10, 9.60]	9.40 [9.10, 9.53]	9.20 [9.00, 9.50]	9.50 [9.35, 9.60]
Corrected Ca, mg/dL, median [IQR] Reference value: 8.5–10.2 mg/dL	8.80 [8.60, 9.10]	8.80 [8.60, 9.10]	8.80 [8.60, 9.10]	8.80 [8.70, 8.97]
Serum albumin, g/dL, median [IQR] Reference value: 3.8–5.2 g/dL	4.50 [4.30, 4.70]	4.50 [4.40, 4.70]	4.50 [4.20, 4.60]	4.60 [4.47, 4.70]
Serum iPTH, pg/mL, median [IQR] Reference value: 10–65 pg/mL	65.00 [48.00, 91.00]	45.00 [28.50, 52.00]	87.00 [75.20, 119.00]	104.00 [50.25, 178.25]
TmP/GFR, mg/dL, median [IQR] Reference value: 2.3–4.3 mg/dL	1.67 [1.34, 1.84]	1.79 [1.56, 2.14]	1.43 [1.19, 1.69]	1.65 [1.47, 1.69]
Serum 25OHD, ng/mL, median [IQR] Deficiency: < 20 ng/mL	16.00 [13.00, 21.00]	17.40 [14.50, 23.00]	15.00 [13.00, 19.00]	19.50 [15.50, 23.25]
Serum 25OHD < 5 ng/mL, n (%)	0 (0.0)	0 (0.0)	0 (0.0)	0 (0.0)
Serum 25OHD 5 to < 20 ng/mL, n (%)	49 (71.0)	20 (62.5)	27 (81.8)	2 (50.0)
Serum 25OHD ≥ 20 ng/mL, n (%)	20 (29.0)	12 (37.5)	6 (18.2)	2 (50.0)
Serum 1,25(OH)2D, pg/mL, median [IQR] Reference value: 20–60 pg/mL	40.20 [24.14, 54.40]	37.50 [26.31, 55.52]	40.90 [26.00, 52.30]	44.55 [21.67, 67.53]
U-Ca/Cr ratio, mg/gCr, median [IQR] Reference value: < 0.3 mg/gCr	0.08 [0.04, 0.14]	0.11 [0.07, 0.16]	0.07 [0.04, 0.09]	0.04 [0.02, 0.11]
Intact FGF23, pg/mL, median [IQR] FGF-related hypophosphatemia: > 30 pg/mL	168.00 [109.00, 387.50]	134.00 [88.00, 295.75]	168.00 [114.00, 427.00]	562.50 [414.75, 1441.00]
Serum creatinine, mg/dL, median [IQR] Reference value: 0.61–1.04 mg/dL for men, 0.47–0.79 mg/dL for women	0.55 [0.46, 0.69]	0.52 [0.46, 0.70]	0.55 [0.46, 0.65]	0.93 [0.80, 1.36]

*Ca* calcium, *FGF23* fibroblast growth factor-23, *iPTH* intact parathyroid hormone, *IQR* interquartile range, *TmP/GFR* ratio of the maximum rate of tubular phosphate reabsorption to the glomerular filtration rate, *U-Ca/Cr ratio* urinary calcium creatinine ratio, *1,25(OH)2D* 1,25-dihydroxyvitamin D

Online Resource 1 shows the characteristics of patients with XLH by sex. The percentage of men with secondary and tertiary hyperparathyroidism was 54.2% (13/24) and 8.3% (2/24), respectively, while that of women was 44.4% (20/45) and 4.4% (2/45), respectively.

### Treatment Status

Ongoing treatments for patients with non-hyperparathyroidism, secondary hyperparathyroidism, and tertiary hyperparathyroidism consisted mostly of active vitamin D (30 [93.8%], 25 [75.8%], and 3 [75.0%], respectively) and oral phosphate (21 [65.6%], 23 [69.7%], and 4 [100.0%], respectively). Only two patients, one with secondary hyperparathyroidism and one with tertiary hyperparathyroidism, had been treated with cinacalcet; none had received evocalcet. Four patients with tertiary hyperparathyroidism underwent surgical procedures, including total parathyroidectomy, parathyroidectomy with autologous transplantation, or total parathyroidectomy with autologous transplantation.

### Renal Dysfunction and Nephrocalcinosis in Hyperparathyroidism

Table 2 shows the association of comorbid hyperparathyroidism with renal function and nephrocalcinosis. Patients with tertiary hyperparathyroidism had numerically lower median [IQR] eGFR (60.86 [52.61, 61.76]) mL/min/1.73 m^2^ than patients with non-hyperparathyroidism and secondary hyperparathyroidism. Regarding CKD stage, most patients with non-hyperparathyroidism and secondary hyperparathyroidism had Stage 1–2 CKD (Stage 1, 81.2% [n = 26] and Stage 2, 15.6% [n = 5]; Stage 1, 81.8% [n = 27] and Stage 2, 9.1% [n = 3], respectively), and most patients with tertiary hyperparathyroidism had Stage 1–2 CKD (75.0% [n = 3]) and Stage 3 CKD (25.0% [n = 1]).

**Table 2:** Renal function and nephrocalcinosis by hyperparathyroidism classification

	Overall	Non-hyperparathyroidism	Secondary hyperparathyroidism	Tertiary hyperparathyroidism
Renal function				
n (%)	69 (100)	32 (46.4)	33 (47.8)	4 (5.8)
eGFR (mL/min/1.73 m^2^), median, [IQR]	113.53 [92.06, 133.94]	114.93 [102.82, 125.11]	117.42 [97.87, 134.86]	60.86 [52.61, 61.76]
CKD stage, n (%)				
Stage 1 (eGFR ≥ 90)	53 (76.8)	26 (81.2)	27 (81.8)	0 (0.0)
Stage 2 (eGFR 60–89)	11 (15.9)	5 (15.6)	3 (9.1)	3 (75.0)
Stage 3a (eGFR 45–59)	2 (2.9)	1 (3.1)	1 (3.0)	0 (0.0)
Stage 3b (eGFR 30–44)	1 (1.4)	0 (0.0)	0 (0.0)	1 (25.0)
Stage 4 (eGFR 15–29)	2 (2.9)	0 (0.0)	2 (6.1)	0 (0.0)
Stage 5 (eGFR < 15)	0 (0.0)	0 (0.0)	0 (0.0)	0 (0.0)
Nephrocalcinosis on renal ultrasound				
n	27	14	10	3
Grade, n (%)				
Grade 1	8 (29.6)	6 (42.9)	2 (20.0)	0.0 (0)
Grade 2	5 (18.5)	2 (14.3)	3 (30.0)	0.0 (0)
Grade 3	7 (25.9)	3 (21.4)	3 (30.0)	1 (33.3)
Grade 4	6 (22.2)	3 (21.4)	2 (20.0)	1 (33.3)
Grade 5	1 (3.7)	0.0 (0)	0.0 (0)	1 (33.3)

Data are number (n) and percentage (%)

*CKD* chronic kidney disease, *eGFR* estimated glomerular filtration rate, *IQR* interquartile range

Grade 1–2 nephrocalcinosis on renal ultrasound tended to be more common among patients with non-hyperparathyroidism (Grade 1, 42.9% [n = 6] and Grade 2, 14.3% [n = 2]) and secondary hyperparathyroidism (Grade 1, 20% [n = 2] and Grade 2, 30% [n = 3]), whereas Grade ≥ 3 nephrocalcinosis on renal ultrasound was more common among those with tertiary hyperparathyroidism (Grade 3, 33.3% [n = 1]; Grade 4, 33.3% [n = 1]; and Grade 5, 33.3% [n = 1]). Renal function and nephrocalcinosis by hyperparathyroidism classification and sex are shown in Online Resource 2.

**Fig. 2:**
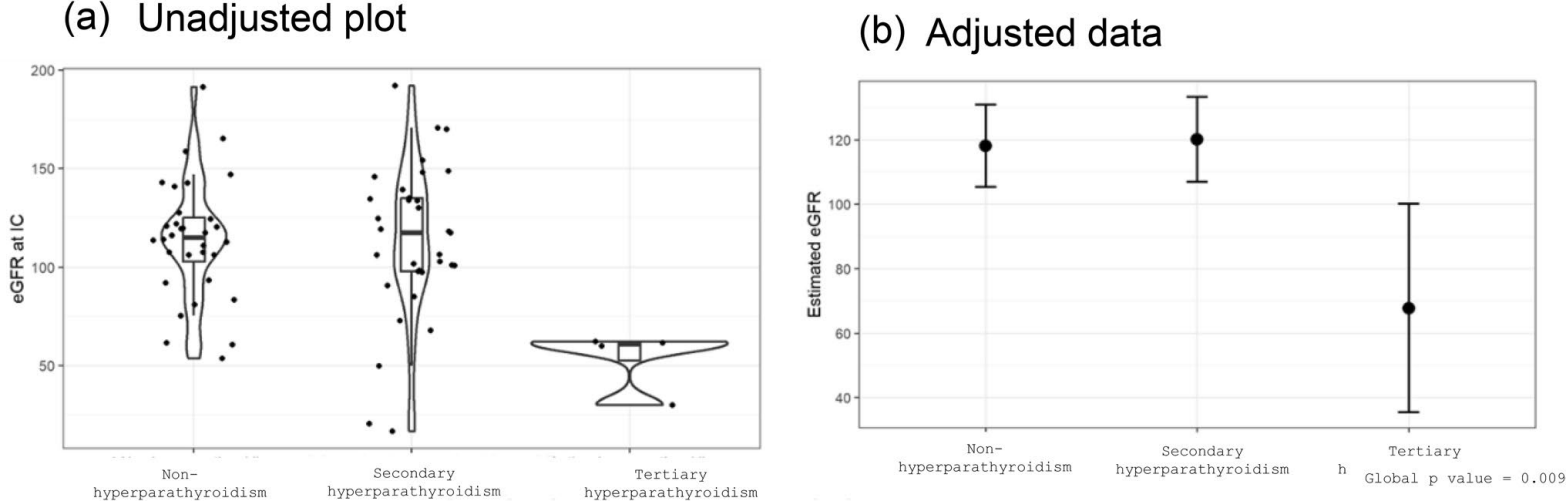
Multivariable linear regression model of renal function by hyperparathyroidism status. **a** Unadjusted; **b** Adjusted by age, sex, and nationality. *eGFR* estimated glomerular filtration rate; *IC* informed consent

Figure 2a shows eGFR by hyperparathyroidism status, and Fig. 2b shows the eGFR after adjustment for age, sex, and country. The eGFR values significantly differed according to the hyperparathyroidism status (global p value = 0.009), and patients with tertiary hyperparathyroidism had the lowest eGFR values. The differences in eGFR between secondary and non-hyperparathyroidism, tertiary and non-hyperparathyroidism, and tertiary and secondary hyperparathyroidism were 2.011 (95% confidence interval [CI]: − 13.465, 17.487), − 50.365 (95% CI: − 83.779, − 16.95), and − 52.376 (95% CI: − 85.304, − 19.447), respectively. The association between eGFR by hyperparathyroidism type and sex is shown in Online Resource 3.

## Discussion

This study analyzed patient data from the SUNFLOWER study to investigate the treatment status and prevalence of comorbid hyperparathyroidism and its association with renal dysfunction in patients with XLH in Japan and South Korea. This analysis is important because the prevalence of hyperparathyroidism and its association with renal dysfunction in Japan and South Korea have been unknown for patients with XLH.

The median age was the highest in the patient group with tertiary hyperparathyroidism, followed by secondary hyperparathyroidism and then non-hyperparathyroidism (aged 45, 34, and 30, respectively). The year from diagnosis of XLH was 50.63 years in patients with tertiary hyperparathyroidism, which was longer than 10.84 years in patients with secondary hyperparathyroidism and 13.18 years in patients without hyperparathyroidism.

This study showed that 53.6% of patients with XLH in Japan and South Korea have hyperparathyroidism. The prevalence of secondary hyperparathyroidism was 47.8%, and that of tertiary hyperparathyroidism was 5.8%. At the time of informed consent, most patients were receiving conventional treatments such as oral phosphate therapy or active vitamin D. Patients with tertiary hyperparathyroidism had a lower eGFR and higher prevalence of nephrocalcinosis and tended to have a more advanced CKD stage than patients in the other two groups.

In the past, several studies have suggested a relationship between the risk of developing secondary/tertiary hyperparathyroidism in patients with XLH with or without conventional therapy [1, 14–19, 21–23]. In the present study, it is possible that the difference in the duration of treatment led to the risk of tertiary hyperparathyroidism because many patients were receiving conventional therapy with oral phosphate or active vitamin D at the time of informed consent.

In a previous study by DeLacey et al. [22], it was found that hyperparathyroidism can increase the risk of renal dysfunction. Our study also supports this finding, as we observed a similar trend. Our results suggest that patients with tertiary hyperparathyroidism have a higher prevalence of nephrocalcinosis and tend to have a higher CKD stage than those without tertiary hyperparathyroidism. Additionally, we found that renal function (based on eGFR) significantly differed according to hyperparathyroidism status, and patients with tertiary hyperparathyroidism had a lower eGFR than those without it. The study by DeLacey et al. also revealed that a patient with tertiary hyperparathyroidism tended to relapse to the same condition after parathyroidectomy. Still, this tendency was not confirmed because of the small numbers in the current study. These findings indicate that tertiary hyperparathyroidism may be the underlying cause of renal dysfunction in patients with XLH.

In addition to CKD, there are other factors contributing to hyperparathyroidism. Among them, the present study focused on (1) vitamin D deficiency, (2) the use of medications that significantly affect mineral metabolism, such as loop diuretics, (3) the use of antiresorptive drugs such as denosumab or bisphosphonates, and (4) primary or secondary aldosteronism. In this study, no patients had severe vitamin D deficiency (25(OH)D level < 5 ng/mL). Of note, there are two definitions for “severe vitamin D deficiency”: one is 25(OH)D level < 5 ng/mL, and the other is 25(OH)D level < 10 ng/mL. Although the second definition may be more common, we adopted the first definition according to Gani et al. [33]. Among the non-hyperparathyroidism, secondary hyperparathyroidism, and tertiary hyperparathyroidism groups, there were no notable differences in the prevalence of vitamin D deficiency, defined as 25(OH)D levels < 20 ng/mL. These results suggest that the impact of low 25(OH)D levels on the outcomes might be small. We also evaluated the prescription of loop diuretics or thiazide diuretics and confirmed that none of the patients were prescribed these medications. Unfortunately, information regarding antiresorptive drugs was not obtained in the present study. Additionally, primary or secondary aldosteronism, which is not a known complication associated with XLH, could not be evaluated or excluded in this study owing to a lack of relevant data.

The relationship between conventional therapy and the risk of developing secondary/tertiary hyperparathyroidism in patients with XLH may be related to a mechanism of secondary hyperparathyroidism caused by oral phosphate in which PTH rises to suppress a temporary increase in serum phosphate levels, resulting in secondary hyperparathyroidism [18]. If this stimulation of parathyroid glands by oral phosphate is repeated, the parathyroid tissue gradually alters to adenomas, and the patient develops tertiary hyperparathyroidism, a condition similar to primary hyperparathyroidism characterized by continuous inappropriate PTH secretion despite high serum calcium concentration.

Tertiary hyperparathyroidism is the same condition as primary hyperparathyroidism, except that the phase of secondary hyperparathyroidism is identified before the onset of the disease. It is known that renal dysfunction sometimes occurs in primary hyperparathyroidism secondary to excess calcium and phosphate urinary excretion [34]. Therefore, in treating patients with XLH, the dosage of oral phosphate should be adjusted within an appropriate range when using conventional therapy, based on calcium and PTH levels, to avoid progression from secondary hyperparathyroidism to tertiary hyperparathyroidism. Some physicians tend to increase oral phosphate too much to normalize serum phosphate levels. It should be noted that serum phosphate levels only increase for 1–2 h after administration of oral phosphate and that serum phosphate levels before oral administration are trough values [35].

Conventional therapy should not be used to normalize serum phosphate levels. Instead, it should be used to improve actual pseudo-fractures or improve biochemical markers (alkaline phosphatase or bone-specific alkaline phosphatase) to indicate treatment efficacy [1].

In cases where tertiary hyperparathyroidism does occur, parathyroidectomy should be undertaken. Furthermore, as mentioned above, patients with XLH who develop tertiary hyperparathyroidism once are prone to recurrent tertiary hyperparathyroidism after parathyroidectomy, and if conventional therapy is continued thereafter, the dosage should be carefully re-set, and calcium, PTH, and creatinine should be carefully monitored [22].

This study had some limitations. As this was a non-randomized observational study, the results may be influenced by selection bias and confounding variables. Although statistical methods were used to adjust the results, only measurable items can be controlled. There were not enough data available to assess the impact of active vitamin D and oral phosphate doses and the duration of this conventional treatment on the development of secondary and tertiary hyperparathyroidism and nephrocalcinosis. Compliance with the prescribed treatment and dosage is crucial for managing hyperparathyroidism. However, although dosing data were collected as far back as possible, the analysis was challenging because of the limited availability of clinical records and lack of precise information on the exact dose and duration of administration for many of the patients. Some patients had received treatment from multiple medical institutions for decades, making it challenging to obtain comprehensive records. As a result, we were unable to determine whether the development of hyperparathyroidism and nephrocalcinosis was related to the doses of oral phosphate and active vitamin D or the duration of administration. The present study was based on data collected at the time of enrollment, with hyperparathyroidism criteria also determined using test values obtained at that time. Consequently, the persistence of elevated iPTH levels over time is unknown. Because the SUNFLOWER study is a 10-year observational study running from 2018 to 2028, future analyses related to drug dosages and hyperparathyroidism may be considered. In addition, all patients with tertiary hyperparathyroidism in this study had previously undergone parathyroidectomy (specific details regarding the procedures were not obtained), and it was difficult to obtain data before the procedure. Therefore, to avoid misunderstanding, alkaline phosphatase and bone-specific alkaline phosphatase were not included.

In conclusion, among this cohort of patients with XLH, the prevalence of secondary hyperparathyroidism was 47.8%, and that of tertiary hyperparathyroidism was 5.8%. Ongoing treatment status showed that patients with secondary hyperparathyroidism and tertiary hyperparathyroidism were primarily treated with active vitamin D and oral phosphate. Calcimimetics were seldom used, with only two patients receiving cinacalcet. In contrast with non-hyperparathyroidism and secondary hyperparathyroidism groups, patients diagnosed with XLH and tertiary hyperparathyroidism exhibited a lower eGFR, a higher prevalence of nephrocalcinosis, and a tendency towards a higher CKD stage. Our study highlights the prevalence of comorbid hyperparathyroidism and its association with renal dysfunction in patients with XLH through a large-scale observational study in Asia. Despite some limitations, the findings can be useful for clinicians to provide a better clinical procedure for patients with XLH.

## Supplementary Information

Below is the link to the electronic supplementary material.

Supplementary file1 (PDF 345 KB)—Online Resource 1. Characteristics of patients with XLH by sex. Online Resource 2. Renal function and nephrocalcinosis by hyperparathyroidism classification and sex. Online Resource 3. Association between eGFR at informed consent by hyperparathyroidism type and sex. Abbreviations: eGFR, estimated glomerular filtration rate; IC, informed consent.

## Data Availability

Research data, including participant data, the statistical analysis plan, and informed consent forms, are not shared. The study protocol has been previously published.

## References

[1] Carpenter TO, Imel EA, Holm IA, Jan de Beur SM, Insogna KL (2011) A clinician’s guide to X-linked hypophosphatemia. J Bone Miner Res 26(7):1381–1388. 10.1002/jbmr.340.Erratum.In:JBoneMinerRes2015;30:394 21538511 10.1002/jbmr.340 PMC3157040

[2] Linglart A, Biosse-Duplan M, Briot K, Chaussain C, Esterle L, Guillaume-Czitrom S, Kamenicky P, Nevoux J, Prié D, Rothenbuhler A, Wicart P, Harvengt P (2014) Therapeutic management of hypophosphatemic rickets from infancy to adulthood. Endocr Connect 4(3):R13-30. 10.1530/EC-13-0103 10.1530/EC-13-0103 PMC3959730 24550322

[3] Munns CF, Yoo HW, Jalaludin MY, Vasanwala R, Chandran M, Rhee Y, But WM, Kong AP, Su PH, Numbenjapon N, Namba N, Imanishi Y, Clifton-Bligh RJ, Luo X, Xia W (2023) Asia-Pacific consensus recommendations on X-linked hypophosphatemia: diagnosis, multidisciplinary management, and transition from pediatric to adult care. JBMR Plus 7:e10744. 10.1002/jbm4.10744 37283655 10.1002/jbm4.10744 PMC10241092

[4] Trombetti A, Al-Daghri N, Brandi ML, Cannata-Andía JB, Cavalier E, Chandran M, Chaussain C, Cipullo L, Cooper C, Haffner D, Harvengt P, Harvey NC, Javaid MK, Jiwa F, Kanis JA, Laslop A, Laurent MR, Linglart A, Marques A, Mindler GT, Minisola S, Yerro MCP, Rosa MM, Seefried L, Vlaskovska M, Zanchetta MB, Rizzoli R (2022) Interdisciplinary management of FGF23-related phosphate wasting syndromes: a consensus statement on the evaluation, diagnosis and care of patients with X-linked hypophosphataemia. Nat Rev Endocrinol 18:366–384. 10.1038/s41574-022-00662-x 35484227 10.1038/s41574-022-00662-x

[5] Haffner D, Emma F, Eastwood DM, Duplan MB, Bacchetta J, Schnabel D, Wicart P, Bockenhauer D, Santos F, Levtchenko E, Harvengt P, Kirchhoff M, Di Rocco F, Chaussain C, Brandi ML, Savendahl L, Briot K, Kamenicky P, Rejnmark L, Linglart A (2019) Clinical practice recommendations for the diagnosis and management of X-linked hypophosphataemia. Nat Rev Nephrol 5:435–455. 10.1038/s41581-019-0152-5 10.1038/s41581-019-0152-5 PMC7136170 31068690

[6] Endo I, Fukumoto S, Ozono K, Namba N, Inoue D, Okazaki R, Yamauchi M, Sugimoto T, Minagawa M, Michigami T, Nagai M, Matsumoto T (2015) Nationwide survey of fibroblast growth factor 23 (FGF23)-related hypophosphatemic diseases in Japan: prevalence, biochemical data and treatment. Endocr J 62:811–816. 10.1507/endocrj.EJ15-0275 26135520 10.1507/endocrj.EJ15-0275

[7] Shimada T, Hasegawa H, Yamazaki Y, Muto T, Hino R, Takeuchi Y, Fujita T, Nakahara K, Fukumoto S, Yamashita T (2004) FGF-23 is a potent regulator of vitamin D metabolism and phosphate homeostasis. J Bone Miner Res 19:429–345. 10.1359/JBMR.0301264 15040831 10.1359/JBMR.0301264

[8] Santos F, Fuente R, Mejia N, Mantecon L, Gil-Peña H, Ordoñez FA (2013) Hypophosphatemia and growth. Pediatr Nephrol 28:595–603. 10.1007/s00467-012-2364-9 23179196 10.1007/s00467-012-2364-9

[9] Zivičnjak M, Schnabel D, Billing H, Staude H, Filler G, Querfeld U, Schumacher M, Pyper A, Schröder C, Brämswig J, Haffner D, Hypophosphatemic Rickets Study Group of Arbeitsgemeinschaft für Pädiatrische Endokrinologie and Gesellschaft für Pädiatrische Nephrologie (2011) Age-related stature and linear body segments in children with X-linked hypophosphatemic rickets. Pediatr Nephrol 26:223–231. 10.1007/s00467-010-1705-9 21120538 10.1007/s00467-010-1705-9

[10] Skrinar A, Dvorak-Ewell M, Evins A, Macica C, Linglart A, Imel EA, Theodore-Oklota C, San Martin J (2019) The lifelong impact of X-linked hypophosphatemia: results from a burden of disease survey. J Endocr Soc 3:1321–1334. 10.1210/js.2018-00365 31259293 10.1210/js.2018-00365 PMC6595532

[11] Ito N, Kang HG, Nishida Y, Evins A, Skrinar A, Cheong HI (2022) Burden of disease of X-linked hypophosphatemia in Japanese and Korean patients: a cross-sectional survey. Endocr J 69:373–383. 10.1507/endocrj.EJ21-0386 34732603 10.1507/endocrj.EJ21-0386

[12] Fukumoto S, Ozono K, Michigami T, Minagawa M, Okazaki R, Sugimoto T, Takeuchi Y, Matsumoto T (2015) Pathogenesis and diagnostic criteria for rickets and osteomalacia—proposal by an expert panel supported by Ministry of Health, Labour and Welfare, Japan, The Japanese Society for Bone and Mineral Research and The Japan Endocrine Society. Endocr J 62:665–671. 10.1507/endocrj.EJ15-0289 26156530 10.1507/endocrj.EJ15-0289

[13] Imel EA, DiMeglio LA, Hui SL, Carpenter TO, Econs MJ (2010) Treatment of X-linked hypophosphatemia with calcitriol and phosphate increases circulating fibroblast growth factor 23 concentrations. J Clin Endocrinol Metab 95:1846–1850. 10.1210/jc.2009-1671 20157195 10.1210/jc.2009-1671 PMC2853995

[14] Alon U, Newsome H, Chan JC (1948) Hyperparathyroidism in patients with X-linked dominant hypophosphatemic rickets—application of the calcium infusion test as an indicator for parathyroidectomy. Int J Pediatr Nephrol 5:39–43 6609135

[15] Rivkees SA, el-Hajj-Fuleihan G, Brown EM, Crawford JD (1992) Tertiary hyperparathyroidism during high phosphate therapy of familial hypophosphatemic rickets. J Clin Endocrinol Metab 75:1514–1518. 10.1210/jcem.75.6.1464657 1464657 10.1210/jcem.75.6.1464657

[16] Carpenter TO, Mitnick MA, Ellison A, Smith C, Insogna KL (1994) Nocturnal hyperparathyroidism: a frequent feature of X-linked hypophosphatemia. J Clin Endocrinol Metab 78:1378–1383. 10.1210/jcem.78.6.8200940 8200940 10.1210/jcem.78.6.8200940

[17] Knudtzon J, Halse J, Monn E, Nesland A, Nordal KP, Paus P, Seip M, Sund S, Sødal G (1995) Autonomous hyperparathyroidism in X-linked hypophosphataemia. Clin Endocrinol (Oxf) 42:199–203. 10.1210/10.1111/j.1365-2265.1995.tb01863.x 7704964 10.1111/j.1365-2265.1995.tb01863.x

[18] Mäkitie O, Kooh SW, Sochett E (2003) Prolonged high-dose phosphate treatment: a risk factor for tertiary hyperparathyroidism in X-linked hypophosphatemic rickets. Clin Endocrinol 58:163–168. 10.1046/j.1365-2265.2003.01685.x 10.1046/j.1365-2265.2003.01685.x 12580931

[19] Carpenter TO, Olear EA, Zhang JH, Ellis BK, Simpson CA, Cheng D, Gundberg CM, Insogna KL (2014) Effect of paricalcitol on circulating parathyroid hormone in X-linked hypophosphatemia: a randomized, double-blind, placebo-controlled study. J Clin Endocrinol Metab 99:3103–3111. 10.1210/jc.2014-2017 25029424 10.1210/jc.2014-2017 PMC4154090

[20] Rafaelsen S, Johansson S, Ræder H, Bjerknes R (2016) Hereditary hypophosphatemia in Norway: a retrospective population-based study of genotypes, phenotypes, and treatment complications. Eur J Endocrinol 174:125–136. 10.1530/EJE-15-0515 26543054 10.1530/EJE-15-0515 PMC4674593

[21] Lecoq AL, Chaumet-Riffaud P, Blanchard A, Dupeux M, Rothenbuhler A, Lambert B, Durand E, Boros E, Briot K, Silve C, Francou B, Piketty M, Chanson P, Brailly-Tabard S, Linglart A, Kamenický P (2020) Hyperparathyroidism in patients with X-linked hypophosphatemia. J Bone Miner Res 35:1263–1273. 10.1002/jbmr.3992 32101626 10.1002/jbmr.3992

[22] DeLacey S, Liu Z, Broyles A, El-Azab SA, Guandique CF, James BC, Imel EA (2019) Hyperparathyroidism and parathyroidectomy in X-linked hypophosphatemia patients. Bone 127:386–392. 10.1016/j.bone.2019.06.025 31276850 10.1016/j.bone.2019.06.025 PMC6836672

[23] Kubota T, Fukumoto S, Cheong HI, Michigami T, Namba N, Ito N, Tokunaga S, Gibbs Y, Ozono K (2020) Long-term outcomes for Asian patients with X-linked hypophosphataemia: rationale and design of the SUNFLOWER longitudinal, observational cohort study. BMJ Open 10:e036367. 10.1136/bmjopen-2019-036367 32601114 10.1136/bmjopen-2019-036367 PMC7328740

[24] Endo I, Fukumoto S, Ozono K, Namba N, Tanaka H, Inoue D, Minagawa M, Sugimoto T, Yamauchi M, Michigami T, Matsumoto T (2008) Clinical usefulness of measurement of fibroblast growth factor 23 (FGF23) in hypophosphatemic patients: proposal of diagnostic criteria using FGF23 measurement. Bone 42:1235–1239. 10.1016/j.bone.2008.02.014 18396126 10.1016/j.bone.2008.02.014

[25] Wu CJ, Song YM, Sheu WH (2000) Tertiary hyperparathyroidism in X-linked hypophosphatemic rickets. Intern Med 39:468–471. 10.2169/internalmedicine.39.468 10852165 10.2169/internalmedicine.39.468

[26] SRL Inc. Directory. Reference values for iPTH. https://test-directory.srl.info/akiruno/test/detail/001180200. Accessed 13 January 2025

[27] SRL Inc. Directory. Reference values for Ca. https://test-directory.srl.info/akiruno/test/detail/004080200. Accessed 13 January 2025

[28] Payne RB, Little AJ, Williams RB, Milner JR (1973) Interpretation of serum calcium in patients with abnormal serum proteins. Br Med J 4:643–646. 10.1136/bmj.4.5893.643 4758544 10.1136/bmj.4.5893.643 PMC1587636

[29] Patriquin H, Robitaille P (1986) Renal calcium deposition in children: sonographic demonstration of the Anderson-Carr progression. AJR Am J Roentgenol 146:1253–1256. 10.2214/ajr.146.6.1253 3518370 10.2214/ajr.146.6.1253

[30] Harada D, Ueyama K, Oriyama K, Ishiura Y, Kashiwagi H, Yamada H, Seino Y (2021) Switching from conventional therapy to burosumab injection has the potential to prevent nephrocalcinosis in patients with X-linked hypophosphatemic rickets. J Pediatr Endocrinol Metab 34:791–798. 10.1515/jpem-2020-0734 33837680 10.1515/jpem-2020-0734

[31] Japanese Society of Nephrology (2019) Essential points from evidence-based clinical practice guidelines for chronic kidney disease 2018. Clin Exp Nephrol 23:1–15. 10.1007/s10157-018-1648-1 30506489 10.1007/s10157-018-1648-1 PMC6344397

[32] Barth JH, Jones RG, Payne RB (2000) Calculation of renal tubular reabsorption of phosphate: the algorithm performs better than the nomogram. Ann Clin Biochem 37:79–81. 10.1258/0004563001901371 10672378 10.1258/0004563001901371

[33] Gani LU, How CH (2015) PILL series vitamin D deficiency. Singapore Med J 56:433–436. 10.11622/smedj.2015119 26311908 10.11622/smedj.2015119 PMC4545131

[34] Insogna KL (2018) Primary hyperparathyroidism. N Engl J Med 37:1050–1059. 10.1056/NEJMcp1714213 10.1056/NEJMcp1714213 30207907

[35] Bettinelli A, Bianchi ML, Mazzucchi E, Gandolini G, Appiani AC (1991) Acute effects of calcitriol and phosphate salts on mineral metabolism in children with hypophosphatemic rickets. J Pediatr 118:372–376. 10.1016/s0022-3476(05)82149-3 1847972 10.1016/s0022-3476(05)82149-3

