# Supplementary material for: Prevalence of Comorbid Hyperparathyroidism and Its Association with Renal Dysfunction in Asian Patients with X-Linked Hypophosphatemic Rickets/Osteomalacia
Source: Calcif Tissue Int. 2025 Mar 12;116(1):50. doi: 10.1007/s00223-025-01359-9 (PMC11903609; doi:10.1007/s00223-025-01359-9)

Prevalence of comorbid hyperparathyroidism and its association with renal dysfunction in Asian patients with X-linked hypophosphatemic rickets/osteomalacia

Nobuaki Ito[1,2], Hee Gyung Kang[3], Toshimi Michigami[4], Noriyuki Namba[5], Takuo Kubota[6], Ayumi Shintani[7], Ryota Kawai[7], Daijiro Kabata[7,8], Haruka Ishii[9], Yayoi Nishida[9], Seiji Fukumoto[10], and Keiichi Ozono[11]

[1]Division of Therapeutic Development for Intractable Bone Diseases, Graduate School of Medicine and Faculty of Medicine, The University of Tokyo, Tokyo, Japan

[2]Osteoporosis Center, The University of Tokyo Hospital, Tokyo, Japan

[3]Division of Pediatric Nephrology, Department of Pediatrics, Seoul National University Children's Hospital, Seoul, South Korea

[4]Department of Bone and Mineral Research, Osaka Women's and Children's Hospital, Osaka Prefectural Hospital Organization, Osaka, Japan

[5]Division of Pediatrics and Perinatology, Tottori University Faculty of Medicine, Tottori, Japan

[6]Department of Pediatrics, Osaka University Graduate School of Medicine, Osaka, Japan

[7]Department of Medical Statistics, Osaka Metropolitan University School of Medicine, Osaka, Japan

[8]Center for Mathematical and Data Sciences, Kobe University, Hyogo, Japan

[9]Medical Affairs Department, Kyowa Kirin Co., Ltd., Tokyo, Japan

[10]Tamaki-Aozora Hospital, Tokushima, Japan

[11]Center for Promoting Treatment of Intractable Diseases, ISEIKAI International General Hospital, Osaka, Japan

Corresponding author

Nobuaki Ito, MD, PhD

Address: Division of Therapeutic Development for Intractable Bone Diseases, Graduate School of Medicine and Faculty of Medicine, The University of Tokyo, 7-3-1 Hongo, Bunkyo-ku, Tokyo 113-8655, Japan

Tel: +81-3-3815-5411

Fax: +81-3-5800-9760

E-mail: nobitotky@gmail.com

Online Resource 1. Characteristics of patients with XLH by sex

	Men				Women			
	**Overall**	**Non-hyperparathyroidism**	**Secondary hyperparathyroidism**	**Tertiary hyperparathyroidism**	**Overall**	**Non-hyperparathyroidism**	**Secondary hyperparathyroidism**	**Tertiary hyperparathyroidism**
n (%)	24 (100)	9 (37.5)	13 (54.2)	2 (8.3)	45 (100)	23 (51.1)	20 (44.4)	2 (4.4)
Age, years, median [IQR]	30.00 [26.75, 44.00]	27.00 [18.00, 43.00]	36.00 [29.00, 47.00]	38.50 [32.25, 44.75]	35.00 [23.00, 49.00]	35.00 [22.50, 48.50]	33.50 [26.00, 48.25]	50.50 [44.75, 56.25]
Nationality, South Korea, n (%)	5 (20.8)	3 (33.3)	2 (15.4)	0 (0.0)	9 (20.0)	5 (21.7)	4 (20.0)	0 (0.0)
Height, cm, median [IQR]	156.50 [154.73, 163.33]	156.60 [154.80, 164.70]	155.70 [149.60, 160.40]	159.10 [157.55, 160.65]	146.95 [143.88, 152.35]	147.95 [145.18, 153.17]	145.85 [140.12, 150.33]	148.75 [142.08, 155.42]
Height, Z-score, median [IQR]	-2.45 [-2.75, -1.27]	-2.43 [-2.74, -1.04]	-2.58 [-3.63, -1.77]	-2.00 [-2.27, -1.73]	-2.12 [-2.71, -1.09]	-1.92 [-2.46, -0.94]	-2.33 [-3.42, -1.48]	-1.78 [-3.05, -0.50]
Weight, kg, median [IQR]	56.30 [52.73, 68.00]	61.60 [53.90, 64.30]	54.80 [53.00, 67.80]	66.70 [59.30, 74.10]	49.05 [44.60, 55.32]	48.50 [44.33, 54.42]	49.35 [46.00, 57.55]	44.85 [41.12, 48.57]
Weight, Z-score, median [IQR]	-0.59 [-1.14, 0.71]	0.09 [-0.94, 0.37]	-0.80 [-1.09, 0.69]	0.17 [-0.56, 0.89]	-0.50 [-1.31, 0.41]	-0.59 [-1.36, 0.29]	-0.44 [-1.04, 0.68]	-1.55 [-2.33, -0.78]

Body mass index, kg/m^2, median [IQR]	23.90 [21.15, 25.77]	24.10 [22.90, 25.70]	23.20 [21.20, 25.40]	26.60 [23.15, 30.05]	22.55 [20.48, 25.52]	21.85 [20.40, 24.95]	23.40 [21.32, 27.75]	20.15 [20.02, 20.27]
Body mass index, Z-score, median [IQR]	0.88 [0.06, 1.31]	0.93 [0.61, 1.29]	0.69 [0.09, 1.23]	0.96 [0.24, 1.69]	0.56 [-0.20, 1.39]	0.33 [-0.22, 1.25]	0.83 [0.15, 1.84]	-0.32 [-0.37, -0.26]
PHEX mutation, n (%)	12 (50.0)	4 (44.4)	7 (53.8)	1 (50.0)	24 (53.3)	13 (56.5)	10 (50.0)	1 (50.0)
Age at diagnosis, years, median [IQR]	1.95 [1.27, 19.35]	6.82 [1.79, 19.14]	1.77 [1.34, 29.31]	0.19 [0.09, 0.28]	19.12 [2.10, 38.54]	13.72 [1.36, 43.93]	23.64 [6.64, 34.65]	2.49 [2.49, 2.49]
Time since diagnosis, years, median [IQR]	16.05 [4.43, 28.98]	13.18 [4.27, 19.28]	25.23 [7.19, 31.61]	38.36 [32.23, 44.49]	11.31 [1.33, 24.16]	13.36 [1.70, 20.60]	3.75 [0.86, 28.47]	59.51 [59.51, 59.51]
Ongoing treatment, n (%)								
Oral phosphate	18 (75.0)	6 (66.7)	10 (76.9)	2 (100.0)	30 (66.7)	15 (65.2)	13 (65.0)	2 (100.0)
Active vitamin D	21 (87.5)	9 (100.0)	10 (76.9)	2 (100.0)	37 (82.2)	21 (91.3)	15 (75.0)	1 (50.0)
History of calcimimetics								
Cinacalcet, n (%)	0 (0.0)	0 (0.0)	0 (0.0)	0 (0.0)	2 (4.4)	0 (0.0)	1 (5.0)	1 (50.0)
Evocalcet, n (%)	0 (0.0)	0 (0.0)	0 (0.0)	0 (0.0)	0 (0.0)	0 (0.0)	0 (0.0)	0 (0.0)
Parathyroidectomy	2 (8.3)	0.0 (0)	0.0 (0)	2 (100.0)	2 (4.4)	0.0 (0)	0.0 (0)	100.0 (2)
Serum phosphate, mg/dL, median [IQR]	2.00 [1.80, 2.20]	2.00 [1.90, 2.10]	2.00 [1.80, 2.10]	2.65 [2.58, 2.72]	2.30 [2.00, 2.50]	2.40 [2.10, 2.70]	2.10 [1.95, 2.50]	2.15 [2.08, 2.22]
Reference value: 2.4–4.3								
Serum calcium, mg/dL, median, [IQR]	9.40 [9.25, 9.75]	9.50 [9.30, 9.90]	9.40 [9.10, 9.70]	9.50 [9.45, 9.55]	9.30 [9.00, 9.50]	9.40 [9.10, 9.50]	9.20 [9.00, 9.40]	9.40 [9.30, 9.50]

Corrected Ca, mg/dL, median [IQR]<br>Reference value: 8.5–10.2	8.80 [8.50, 9.10]	9.00 [8.40, 9.10]	8.80 [8.50, 9.10]	8.95 [8.82, 9.07]	8.80 [8.60, 9.00]	8.80 [8.65, 9.05]	8.80 [8.60, 9.03]	8.80 [8.75, 8.85]
Serum albumin, g/dL, median [IQR]<br>Reference value: 3.8–5.2	4.60 [4.50, 4.90]	4.60 [4.50, 4.90]	4.60 [4.20, 4.90]	4.55 [4.48, 4.62]	4.50 [4.30, 4.60]	4.40 [4.30, 4.60]	4.50 [4.18, 4.53]	4.60 [4.55, 4.65]
Serum iPTH, pg/mL, median [IQR]<br>Reference value: 10–65	70.00 [45.00, 97.75]	35.70 [24.00, 48.30]	86.90 [73.00, 141.00]	94.50 [65.25, 123.75]	59.00 [48.00, 87.00]	48.00 [30.00, 53.00]	89.00 [76.55, 116.75]	154.50 [104.75, 204.25]
TmP/GFR, mg/dL, median [IQR]<br>Reference value: 2.3–4.3	1.52 [1.25, 1.69]	1.69 [1.46, 1.93]	1.43 [1.14, 1.53]	1.70 [1.69, 1.71]	1.70 [1.38, 2.01]	1.79 [1.58, 2.27]	1.65 [1.25, 1.83]	1.31 [1.16, 1.47]
Serum 25OHD, ng/mL, median [IQR]<br>Deficiency: <20	17.50 [13.00, 21.75]	18.00 [13.00, 25.00]	16.00 [13.00, 19.00]	19.00 [16.50, 21.50]	16.00 [13.00, 20.00]	17.00 [15.00, 22.50]	14.50 [12.23, 16.38]	19.50 [17.75, 21.25]
Serum 1,25(OH)2D, pg/mL, median [IQR]<br>Reference value: 20–60	39.60 [23.91, 56.20]	51.50 [24.14, 61.50]	39.00 [26.90, 53.60]	45.20 [33.55, 56.85]	40.20 [26.00, 52.30]	37.00 [27.57, 53.05]	42.15 [25.40, 48.17]	44.10 [32.55, 55.65]
U-Ca/Cr ratio, mg/gCr, median [IQR]<br>Reference value: <0.3	0.07 [0.04, 0.09]	0.10 [0.07, 0.16]	0.06 [0.03, 0.08]	0.04 [0.03, 0.04]	0.10 [0.06, 0.16]	0.12 [0.08, 0.16]	0.07 [0.04, 0.12]	0.16 [0.08, 0.23]

Intact FGF23, pg/mL, median [IQR] FGF-related hypophosphatemia: >30	288.00 [146.50, 596.50]	231.00 [139.00, 562.25]	208.00 [147.00, 456.00]	2194.00 [1441.00, 2947.00]	134.00 [88.97, 268.00]	117.50 [87.10, 266.75]	146.00 [102.62, 229.50]	392.50 [370.25, 414.75]
Serum creatinine, mg/dL, median [IQR] Reference value: 0.61–1.04 for men, 0.47–0.79 for women	0.69 [0.58, 0.84]	0.75 [0.58, 0.81]	0.64 [0.57, 0.73]	1.69 [1.36, 2.01]	0.48 [0.43, 0.57]	0.48 [0.45, 0.54]	0.50 [0.43, 0.57]	0.77 [0.75, 0.80]

Ca = calcium, eGFR = estimated glomerular filtration rate, FGF23 = fibroblast growth factor-23, iPTH = intact parathyroid hormone, IQR = interquartile range, TmP/GFR = ratio of the maximum rate of tubular phosphate reabsorption to the glomerular filtration rate, U-Ca/Cr ratio = urinary calcium creatinine ratio, 1,25(OH)2D = 1,25-dihydroxyvitamin D

Online Resource 2. Renal function and nephrocalcinosis by hyperparathyroidism classification and sex

	**Men**				**Women**			
	**Overall**	**Non-hyperparathyroidism**	**Secondary hyperparathyroidism**	**Tertiary hyperparathyroidism**	**Overall**	**Non-hyperparathyroidism**	**Secondary hyperparathyroidism**	**Tertiary hyperparathyroidism**
Renal function								
n (%)	24 (100)	9 (37.5)	13 (54.2)	2 (8.3)	45 (100)	23 (51.1)	20 (44.4)	2 (4.4)
eGFR, median [IQR]	114.292 (82.902, 126.870)	115.937 (83.524, 119.621)	119.099 (97.874, 139.339)	45.088 (37.567, 52.609)	113.526 (97.562, 134.610)	113.916 (106.070, 134.182)	111.782 (98.135, 134.673)	61.921 (61.759, 62.082)
CKD stage, n (%)								
Stage 1 (eGFR ≥90)	17 (71)	6 (67)	11 (85)	0 (0)	36 (80)	20 (87)	16 (80)	0 (0)
Stage 2 (eGFR 60–89)	4 (17)	3 (33)	0 (0)	1 (50)	7 (16)	2 (8.7)	3 (15)	2 (100)
Stage 3a (eGFR 45–59)	1 (4.2)	0 (0)	1 (7.7)	0 (0)	1 (2.2))	1 (4.3)	0 (0)	0 (0)
Stage 3b (eGFR 30–44)	1 (4.2)	0 (0)	0 (0)	1 (50)	0 (0)	0 (0)	0 (0)	0 (0)
Stage 4 (eGFR 15–29)	1 (4.2)	0 (0)	1 (7.7)	0 (0)	1 (2.2)	0 (0)	1 (50)	0 (0)
Stage 5 (eGFR <15)	0 (0)	0 (0)	0 (0)	0 (0)	0 (0)	0 (0)	0 (0)	0 (0)

Nephrocalcinosis on renal ultrasound								
n	11	4	5	2	16	10	5	1
Grade, n (%)								
Grade 1	2 (18)	2 (50)	0 (0)	0 (0)	6 (38)	4 (40)	3 (40)	0 (0)
Grade 2	1 (9.1)	0 (0)	1 (20)	0 (0)	4 (25)	2 (20)	2 (40)	0 (0)
Grade 3	4 (36)	1 (25)	3 (60)	0 (0)	3 (19)	2 (20)	0 (0)	1(100)
Grade 4	3 (27)	1(25)	1 (20)	1 (50)	3 (19)	2 (20)	1 (20)	0 (0)
Grade 5	1 (9.1)	0 (0)	0 (0)	1 (50)	0 (0)	1 (0)	0 (0)	0 (0)

Data are number (n) and percentage (%).

eGFR = estimated glomerular filtration rate, IQR = interquartile range

Online Resource 3. Association between eGFR at informed consent by hyperparathyroidism type and sex

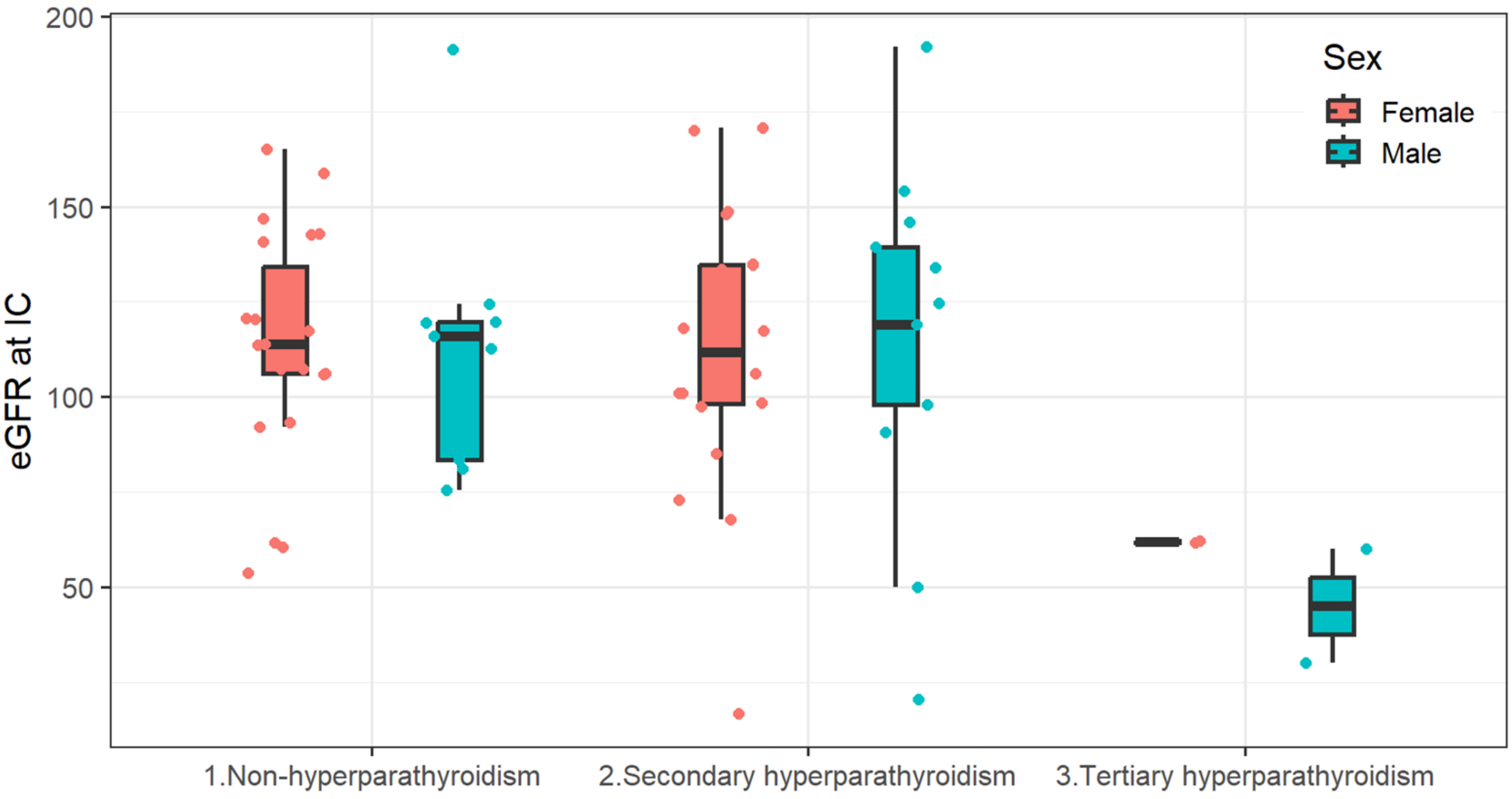

Supplement: Supplementary file 1 — Supplementary file1 (PDF 345 KB)—Online Resource 1. Characteristics of patients with XLH by sex. Online Resource 2. Renal function and nephrocalcinosis by hyperparathyroidism classification and sex. Online Resource 3. Association between eGFR at informed consent by hyperparathyroidism type and sex. Abbreviations: eGFR, estimated glomerular filtration rate; IC, informed consent. [file 223_2025_1359_MOESM1_ESM.pdf]
